# Supplementary material for: Evolutionary bioenergetics of sporulation
Source: Proc Natl Acad Sci U S A. 2026 Feb 6;123(6):e2524274123. doi: 10.1073/pnas.2524274123 (PMC12890906; doi:10.1073/pnas.2524274123)
Supplement: Supplementary file 1 — Appendix 01 (PDF) [file pnas.2524274123.sapp.pdf]

PNAS

Supporting Information for

Evolutionary bioenergetics of sporulation

Canan Karakoç, William R. Shoemaker, and Jay T. Lennon

Corresponding Author: Jay T. Lennon
E-mail: lennonj@iu.edu

This PDF file includes:

Figs. S1 to S3
Tables S1 to S4
SI References

1. Predicting sporulation efficiency in batch culture

There is an increasing appreciation that models with static environments are unlikely to be reflective of the temporally varying environments that microorganisms frequently encounter (1–3). Batch culture models provide a means to incorporate such realism, having been leveraged to investigate microbial physiology (4, 5), ecology (2, 6), and evolution (7, 8). Such models are particularly suited to questions regarding spore-formation, as ecological theory suggests that such life-history strategies are primarily beneficial in time-varying environments (9, 10) and batch culture experimental designs have been used to investigate sporulation efficiency (Table S2).

Our model of microbial growth and spore formation in batch culture was defined as:

$$\frac{dN_v}{dt} = N_v \left[\underbrace{g_v(R)}_{\text{Cell growth}} - \underbrace{f(R) \cdot g_s(R)}_{\text{Spore formation}} \right] \quad [1]$$

$$\frac{dN_s}{dt} = N_v \left[\underbrace{f(R) \cdot g_s(R)}_{\text{Spore formation}} \right] \quad [2]$$

$$\frac{dR}{dt} = -N_v \left[\underbrace{\frac{g_v(R)}{Y_v}}_{\text{Consumption}} + \underbrace{\frac{f(R) \cdot g_s(R)}{Y_s}}_{\text{Consumption}} \right] \quad [3]$$

where the rate of vegetative cellular growth and sporulation were defined as

$$g_v(R) = r_{\text{max}}^{(v)} \frac{R}{R + K_v} \quad [4]$$

$$g_s(R) = r_{\text{max}}^{(s)} \frac{R}{R + K_s} \quad [5]$$

where r_{max} is the maximum growth rate, and K is the half-saturation constant, specifying the value of R where $g(R)$ is half of r_{max}. The rate of spore initiation (i.e., the rate that a cell initiates the process of sporulation) is modeled as the following function:

$$f(R) = \frac{1}{1 + e^{\sigma(R - R_{\text{min}})}} \quad [6]$$

When $\sigma \gg 1$, the function approximates a Heaviside step function (11):

$$f(R) = \begin{cases} 1 & \text{for } R(t) \leq R_{\text{min}} \\ 0 & \text{for } R(t) > R_{\text{min}} \end{cases}$$

We do not consider spore germination as it is unneeded for a single batch culture cycle where resource concentration does not increase. We do not incorporate a death rate term, a choice that reflects the assumption that the death of vegetative cells does not make an appreciable contribution relative to growth and sporulation over the timescale of our defined batch culture cycle. The absence of this term is common in batch culture consumer-resource models (e.g., (6)). The absence of a death rate for endospores reflects their extreme longevity. The absence of a death rate is useful in that it allows us to apply the principle of mass conservation to obtain analytic solutions without the introduction of additional ODEs.

The resource concentration $R(t)$ does not reach negative values because all terms in the above model contain functions that approach zero as $R(t) \to 0$. This is an outcome of the rate of decline of R being proportional to the sum of the fluxes of consumption for 1) cellular growth and 2) spore formation, each of which are dependent on functions of resource concentration (R). So while dR/dt depends on N_v, the functions that N_v is multiplied by are themselves approaching zero from the right hand side.

Batch culture models as defined above do not have a steady-state, defined as a fixed point independent of initial conditions. Instead, variables approach long-term limits that are often dependent on initial conditions. We investigate this limit in our model and leverage the principle of mass conservation to derive sporulation efficiency (ϕ). Here, the sum of the supplied concentration of resources and the concentration of spore and vegetative cell abundances, corrected for yield, sets the total concentration of mass in the system. This initial sum must be equal to the sum at time t.

$$R_0 + \frac{N_v(0)}{Y_v} \equiv R(t) + \frac{N_v(t)}{Y_v} + \frac{N_s(t)}{Y_s} \quad [7]$$

We have specified that no endospores were present at inoculation ($N_s(0) = 0$), and that all resources were supplied at the start of the experiment. These resources decrease in concentration as the concentration of cells and spores saturates, establishing a timescale t_{sat} where the resources are effectively depleted $R(t_{\text{sat}}) \approx 0$. At this timescale, the mass balance relation reduces to the following:

$$R_0 + \frac{N_v(0)}{Y_v} \equiv \frac{N_v(t_{\text{sat}})}{Y_v} + \frac{N_s(t_{\text{sat}})}{Y_s} \tag{8}$$

from which we can rearrange to obtain functions of the abundances of vegetative cells and spores.

$$N_v^{\text{sat}} \equiv N_v(t_{\text{sat}}) = Y_v \left[R_0 + \frac{N_v(0)}{Y_v} - \frac{N_s^{\text{sat}}}{Y_s} \right] \tag{9}$$

$$N_s^{\text{sat}} \equiv N_s(t_{\text{sat}}) = Y_s \left[R_0 + \frac{N_v(0)}{Y_v} - \frac{N_v^{\text{sat}}}{Y_v} \right] \tag{10}$$

Using these stationary values, we can derive a prediction for the fraction of spores in the population, known as sporulation efficiency:

$$\begin{aligned} \phi &\equiv \frac{N_s^{\text{sat}}}{N_s^{\text{sat}} + N_v^{\text{sat}}} \\ &= \left[1 + \frac{N_v^{\text{sat}}}{N_s^{\text{sat}}} \right]^{-1} \\ &= \left[1 + \frac{Y_v}{N_s^{\text{sat}}} \left(R_0 + \frac{N_v(0)}{Y_v} - \frac{N_s^{\text{sat}}}{Y_s} \right) \right]^{-1} \end{aligned} \tag{11}$$

So, we have a prediction of sporulation efficiency that only depends on one observable, the final density of spores N_s^{sat}. An equivalent prediction can be obtained that only relies on N_s^{sat}. This reliance on one unknown is because mass conservation is a linear relation between the number of spores (N_s) and vegetative cells (N_v), meaning that it cannot uniquely determine ϕ. Therefore, we evaluated the effect of energetic costs on the above model ϕ using numerical simulations (described below).

We can also obtain a solution for ϕ within a single batch culture cycle by making additional assumptions. We assume the parameter regime $R_0 \ll K_v$, K_s, linearizing the growth functions $g_v(R)$, $g_s(R)$. In this parameter regime when $\sigma \gg 1$, spores do not form over timescale t_{min}, reducing the system to

$$\frac{dN_v}{dt} = N_v R \frac{r_{\text{max}}^{(v)}}{K_v} \tag{12}$$

$$\frac{dR}{dt} = -N_v R \frac{r_{\text{max}}^{(v)}}{K_v Y_v} \tag{13}$$

The mass balance equation is then for $t \in [0, t_{\text{min}}]$

$$R_0 + \frac{N_v(0)}{Y_v} \equiv R(t) + \frac{N_v(t)}{Y_v} \tag{14}$$

Solving for $N_v(t)$, we remove the dependency on $R(t)$ and obtain

$$\begin{aligned} \frac{dN_v}{dt} &= N_v \left(R_0 + \frac{N_v(0)}{Y_v} - \frac{N_v(t)}{Y_v} \right) \frac{r_{\text{max}}^{(v)}}{K_v} \\ &= N_v \tilde{r}_v \left(1 - \frac{N_v}{\tilde{K}} \right) \end{aligned} \tag{15}$$

where we have defined $\tilde{r}_v \equiv \frac{r_{\text{max}}^{(v)}}{K_v} \left(R_0 + \frac{N_v(0)}{Y_v} \right)$ and $\tilde{K} \equiv Y \left(R_0 + \frac{N_v(0)}{Y_v} \right)$ as the effective growth rate and carrying capacity. We recognize this as the ordinary differential equation (ODE) for logistic growth, the solution of which is:

$$N_v(t) = \frac{\tilde{K}}{1 + \left(\frac{\tilde{K} - N_v(0)}{N_v(0)} \right) e^{-\tilde{r}_v t}} \tag{16}$$

from which one can calculate the number of vegetative cells *before* the onset of sporulation, $N_v(t_{\text{min}})$. After t_{min}, we assume that all vegetative cells initiate sporulation. For $t \in (t_{\text{min}}, t_{\text{sat}}]$, the dynamics are then:

$$\frac{dN_v}{dt} = -N_v R \frac{r_{\max}^{(s)}}{K_s} \tag{17}$$

$$\frac{dN_s}{dt} = N_v R \frac{r_{\max}^{(s)}}{K_s} \tag{18}$$

$$\frac{dR}{dt} = -N_v R \frac{r_{\max}^{(s)}}{K_s Y_s} \tag{19}$$

Looking at this set of equations, we notice that N_v is effectively relabeled as N_v by the consumption of resources over $t \in (t_{\min}, t_{\text{sat}}]$, in contrast to the first period of time $t \in [0, t_{\min}]$ where N_v reproduces by consuming resources. Alternatively stated, N_v contains mass but does not exchange it with R within $t \in (t_{\min}, t_{\text{sat}}]$. This insight allows us to construct a mass balance formula for R that does not contain N_v. The mass balance equation for $t \in (t_{\min}, t_{\text{sat}}]$ is then

$$R_{\min} = R(t) + \frac{N_s(t)}{Y_s} \tag{20}$$

Since $\frac{dN_v}{dt} = -\frac{dN_s}{dt}$, we also obtain the following identity

$$N_v(t_{\min}) = N_v(t) + N_s(t) \tag{21}$$

Using these two identities, we can write

$$N_v(t) = N_v(t_{\min}) - N_s(t) \tag{22}$$

$$R(t) = R_{\min} - \frac{N_s(t)}{Y_s} \tag{23}$$

from which we obtain a single ODE for the number of spores

$$\frac{dN_s}{dt} = \frac{r_{\max}^{(s)}}{K_s} \left(N_v(t_{\min}) - N_s(t)\right) \left(R_{\min} - \frac{N_s(t)}{Y_s}\right) \tag{24}$$

This ODE is biologically meaningful over the domain $0 \leq N_s \leq \min\{N_v(t_{\min}), Y_s R_{\min}\}$. We can solve the ODE using separation of variables

$$\frac{r_{\max}^{(s)}}{K_s}(t - t_{\min}) = \int_{N_s(t_{\min}=0)}^{N_s(t)} \frac{dN'}{\left(N_v(t_{\min}) - N'\right)\left(R_{\min} - \frac{N'}{Y_s}\right)} \tag{25}$$

obtaining

$$N_s(t) = R_{\min} Y_s \cdot \frac{1 - e^{-\tilde{r}(t - t_{\min})}}{1 - \frac{R_{\min} Y_s}{N_v(t_{\min})} e^{-\tilde{r}(t - t_{\min})}} \tag{26}$$

where $\tilde{r} \equiv \frac{r_{\max}^{(s)}}{K_s}\left(\frac{N_v(t_{\min})}{Y_s} - R_{\min}\right)$. This effective rate of sporulation is biologically meaningful when $N_v(t_{\min}) > Y_s R_{\min}$. We combine our results to obtain a measure of sporulation efficiency.

$$\phi(t) = \begin{cases} 0, & 0 \leq t \leq t_{\min}, \\ \dfrac{N_s(t)}{N_v(t) + N_s(t)} = \dfrac{N_s(t)}{N_v(t_{\min})}, & t_{\min} < t \leq t_{\text{sat}}. \end{cases} \tag{27}$$

where $\phi(t_{\text{sat}}) \approx \frac{Y_s R_{\min}}{N_v(t_{\min})}$ in the limit $t_{\text{sat}} - t_{\min} \gg \tilde{r}^{-1}$. This expression demonstrates how sporulation efficiency can decline with increasing sporulation costs (i.e., decreasing yield) in a time-varying environment.

Table S1. Parameter values used in simulations

Variable	Meaning	Value
R_0	Initial resource concentration	$10^3\ \mu g/\mathrm{mL}$
$N_v(0)$	Initial cell density	10^5 cells/mL
$N_s(0)$	Initial spore density	0 spores/mL
$r^{(v)}_{\mathsf{max}}$	Maximum growth rate of cells	$2\ \mathrm{hr}^{-1}$
$r^{(s)}_{\mathsf{max}}$	Maximum rate of spore formation	$1/8\ \mathrm{hr}^{-1}$
K_v	Monod constant for cellular growth	$3.2 \cdot R_0\ \mu g/\mathrm{mL}$
K_s	Monod constant for spore formation	$0.1 \cdot R_0\ \mu g/\mathrm{mL}$
σ	Sharpness of spore formation initiation	$0.1\ \mathrm{mL}/\mu g$
R_{min}	Resource threshold for sporulation	$5\ \mu g/\mathrm{mL}$
Y_v	Cell yield	$\epsilon(5 \cdot 10^{-11})$ cells$/\mu g$
Y_s	Spore yield	$\epsilon(1 \cdot 10^{-11})$ spores$/\mu g$

2. Predicting sporulation efficiency in a chemostat

As a point of comparison, it is worth contrasting the above result with the solution one obtains by using a chemostat instead of a batch culture. This can be accomplished by amending each equation in our batch culture system with 1) a dilution term δ that defines the fraction of total vessel volume that flows in and out of the chemostat per unit time and 2) the concentration of resources supplied to the vessel R_0.

$$\frac{dN_v}{dt} = N_v \left[\underbrace{g_v(R)}_{\text{Cell growth}} - \underbrace{f(R) \cdot g_s(R)}_{\text{Spore formation}} - \underbrace{\delta}_{\text{Dilution}} \right] \quad [28]$$

$$\frac{dN_s}{dt} = N_v \underbrace{f(R) \cdot g_s(R)}_{\text{Spore formation}} - N_s \underbrace{\delta}_{\text{Dilution}} \quad [29]$$

$$\frac{dR}{dt} = \underbrace{\delta R_0}_{\text{Input}} - \underbrace{\frac{N_v g_v(R)}{Y_v}}_{\text{Consumption}} - \underbrace{\frac{N_v f(R) \cdot g_s(R)}{Y_s}}_{\text{Consumption}} - \underbrace{\delta R}_{\text{Dilution}} \quad [30]$$

This model marks a clear and biologically meaningful difference from a model of batch culture dynamics, as there now exists a stationary non-zero resource concentration ($R^* > 0$). We have also assumed that the intrinsic timescale of the system, set by δ^{-1}, is sufficient such that sporulation before washout is feasible, but that sporulation + revival is not. The stationary solution can be obtained by setting the derivatives to zero.

$$\begin{aligned} 0 &= g_v(R^*) - f(R^*) g_s(R^*) - \delta \\ 0 &= N_v^* f(R^*) g_s(R^*) - N_s^* \delta \\ 0 &= \delta R_0 - \delta R^* - \frac{N_v^* g_v(R^*)}{Y_v} - \frac{N_v^* f(R^*) \cdot g_s(R^*)}{Y_s} \end{aligned} \quad [31]$$

We can make analytic progress by assuming that the stationary resource concentration is less than the half-saturation constants, $R^* \ll K_v, K_s$, a limit that can be experimentally obtained by tuning the dilution rate. This limit allows us to make the linear approximation $g(R^*) \approx \frac{R^* r_{\max}}{K}$. Furthermore, if $R^* \ll R_{\min}$. We can further reduce the system by using the Heaviside limit for the rate of spore formation (i.e., $\sigma \gg 1$). Using these approximations, the above equations reduce to the following for $R^* > R_{\min}$

$$\begin{aligned} 0 &= R^* \frac{r^{(v)}_{\max}}{K_v} - \delta \\ 0 &= \delta R_0 - \delta R^* - N_v^* R^* \cdot \frac{1}{Y_v} \frac{r^{(s)}_{\max}}{K_s} \end{aligned} \quad [32]$$

where spore formation is not permitted. For the regime $R^* \leq R_{\min}$

$$\begin{aligned} 0 &= R^* \cdot \frac{r^{(v)}_{\max}}{K_v} - R^* \cdot \frac{r^{(s)}_{\max}}{K_s} - \delta \\ 0 &= N_v^* R^* \cdot \frac{r^{(s)}_{\max}}{K_s} - N_s^* \delta \\ 0 &= \delta R_0 - \delta R^* - N_v^* R^* \cdot \frac{1}{Y_v} \frac{r^{(v)}_{\max}}{K_v} - N_v^* R^* \cdot \frac{1}{Y_s} \frac{r^{(s)}_{\max}}{K_s} \end{aligned} \quad [33]$$

Using these approximations, we obtain the following stationary solutions:

$$R^* = \delta \left[\frac{r_{\max}^{(v)}}{K_v} - \frac{r_{\max}^{(s)}}{K_s} \right]^{-1} \quad [34]$$

$$N_v^* = \frac{\delta(R_0 - R^*)}{R^* \left(\frac{r_{\max}^{(v)}}{Y_v K_v} + \frac{r_{\max}^{(s)}}{Y_s K_s} \right)} \quad [35]$$

$$N_s^* = N_v^* R^* \frac{1}{\delta} \frac{r_{\max}^{(s)}}{K_s} \quad [36]$$

where $R^* > 0$ if $\frac{r_{\max}^{(v)}}{K_v} > \frac{r_{\max}^{(s)}}{K_s}$
From which we obtain the steady-state sporulation efficiency:

$$\begin{aligned} \phi^* &\equiv \frac{N_s^*}{N_s^* + N_v^*} \\ &= \frac{r_{\max}^{(s)} K_v}{r_{\max}^{(v)} K_s} \end{aligned} \quad [37]$$

This result can be interpreted as the ratio of the rates of cellular growth and endospore formation, with yield playing no role. This result means that one would expect energetic costs not to factor into sporulation efficiency, contrasting with the batch culture scenario. This analytic result is consistent with prior experimental efforts to quantify efficiency in a chemostat setting (S1; (12)).

3. Simulating batch culture dynamics

We investigated the validity of our sporulation efficiency predictions across spore formation parameter regimes, focusing on the two terms governing spore formation: the Monod function governing the formation of spores $g_s(R)$ and the rate of initiating spore formation $f(R)$. Our system of equations was solved using the `solve_ivp()` function from SciPy v1.10.1 for a given parameter combination (13), from which sporulation efficiency was calculated. In numerical simulations, the lowest observed resource concentration are effectively zero ($R \approx -10^{-6}$), with small negative values arising due to numerical limitations typical of ODE simulations (e.g., round-off error, solver tolerance).

We found that our predictions tended to fail when the Monod constant is small relative to the concentration of supplied resources (S2), reducing the Monod function to the constant $r_{\max}^{(s)}$. This deviation is consistent with the assumptions in our derivation, as the limit $K_s \ll R_0$ effectively decouples resource consumption from growth. In contrast, our predictions remained consistently accurate across spore formation initiation parameters (S2). This result suggests that details of spore formation initiation may not considerably shape predictions obtained at an extended timescale.

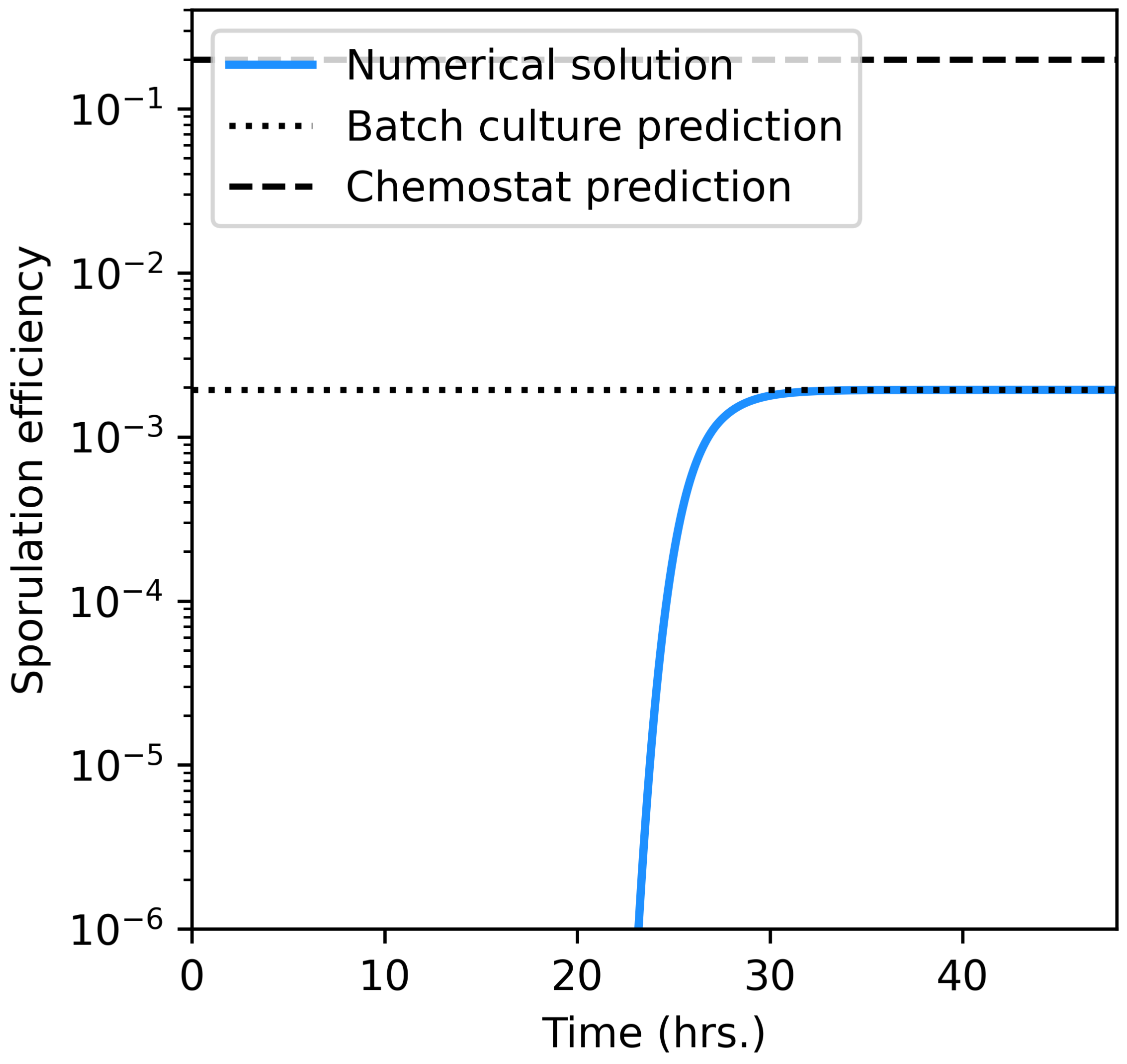

Fig. S1. Our batch culture predictions of sporulation efficiency were validated by obtaining the numerical solution of our system of differential equations. As resources become depleted, predictions that depend on the yields of cells and spores (dashed line) become increasingly accurate. The prediction in the chemostat limit is plotted as a point of comparison.

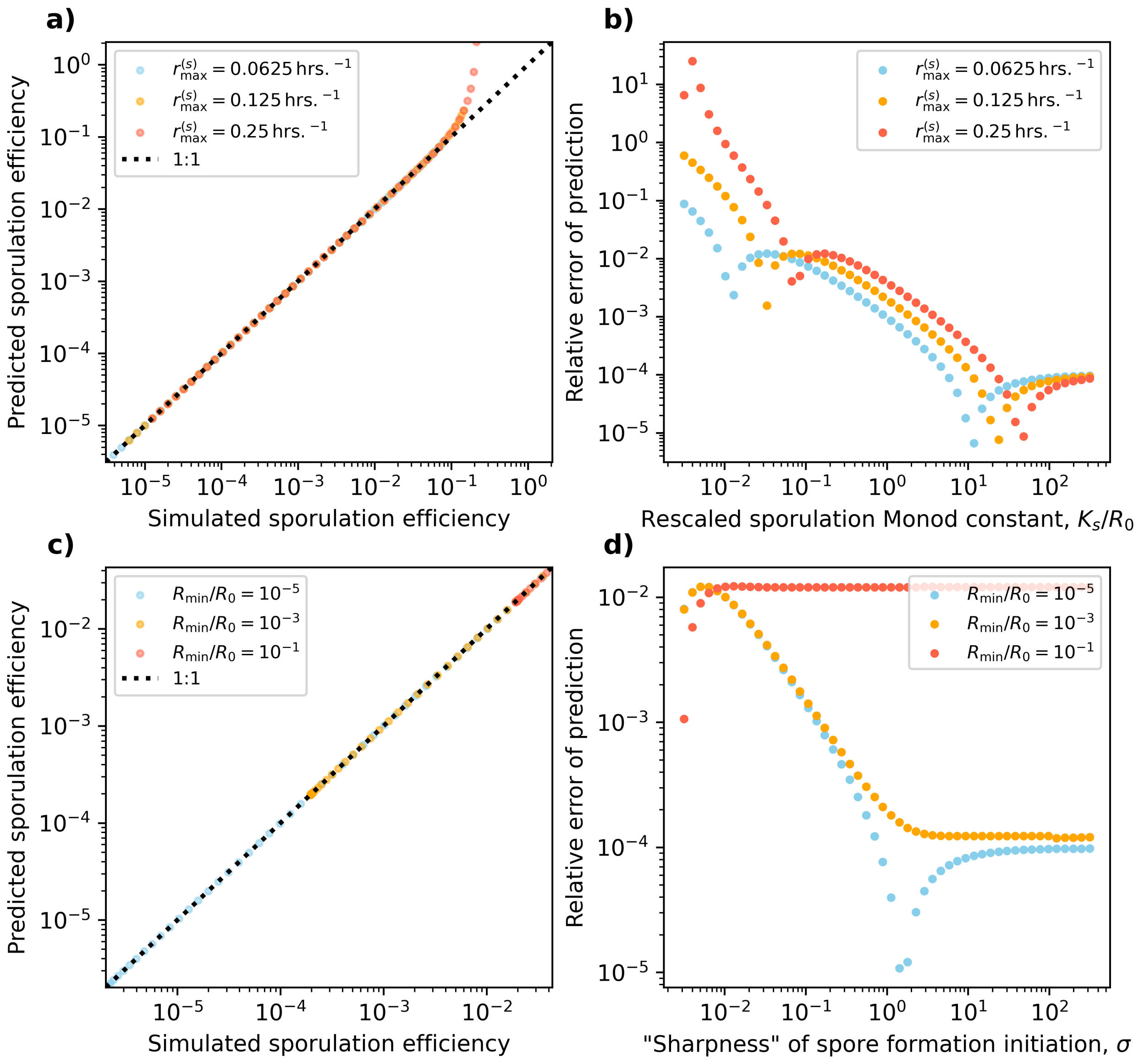

Fig. S2. The effect of parameters controlling spore formation on the validity of sporulation efficiency predictions.

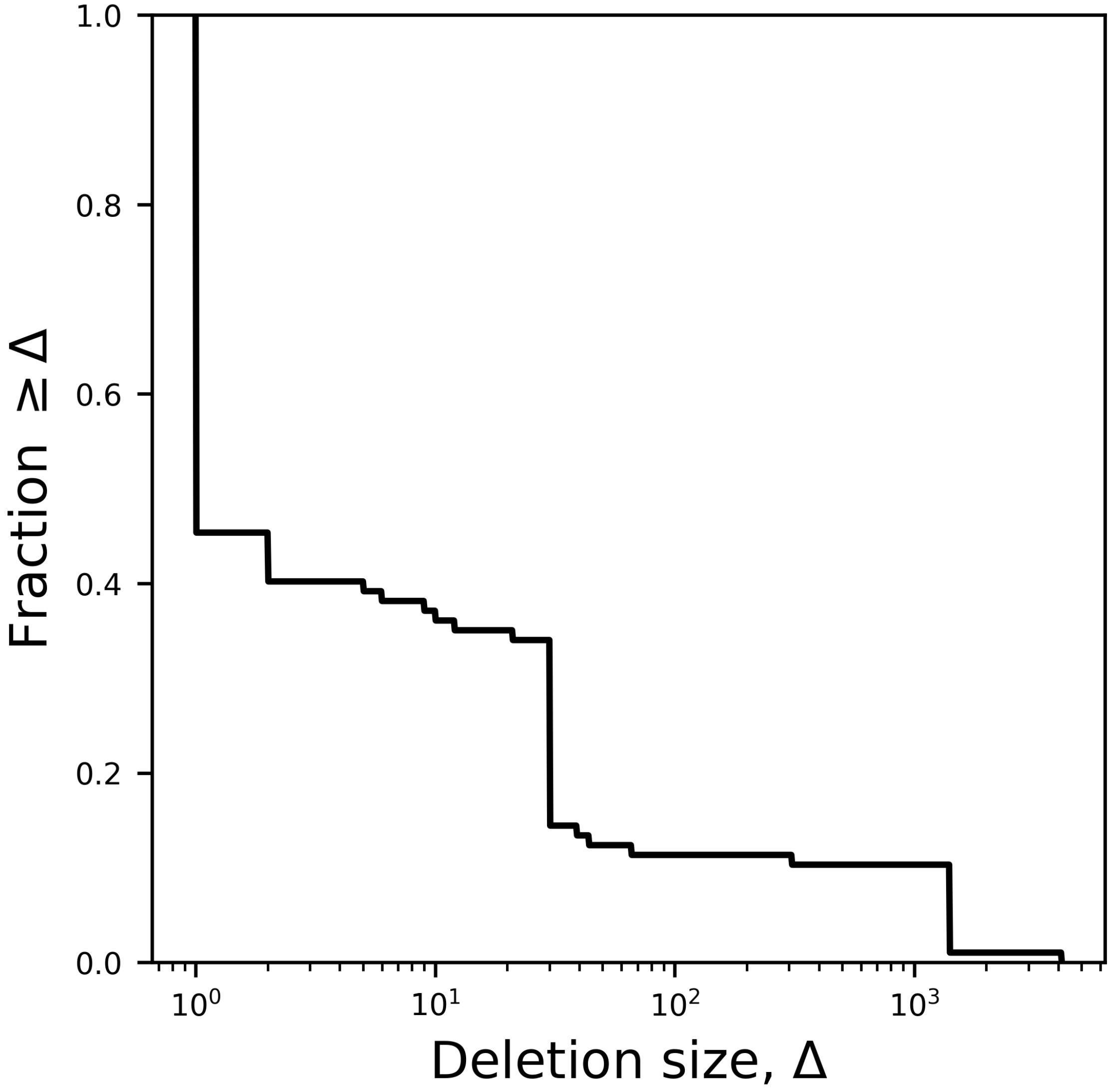

Fig. S3. The empirical distribution of deletion sizes in *Bacillus subtilis*.

Table S2. Reported sporulation efficiencies from the literature.

Publication	Efficiencies (%)	Culture system	DOI
Grossman and Losick 1988	23, 34	Batch (S7 medium, shaking flasks)	doi.org/10.1073/pnas.85.12.4369
Kudo and Horikoshi 1979	0, 1, 5, 10, 15, 30, 35, 50, 55, 70, 95	Batch (AMS medium, flask cultures)	doi.org/10.1080/00021369.1979.10863868
Monteiro et al. 2008	10, 15.3, 16.7, 17, 23.6, 25.6, 40.9, 45.2, 45.9, 46.4, 47.9, 48, 48.8, 50.7, 53.2, 58.8	Batch / fed-batch bioreactor (DSM)	doi.org/10.1021/bp050062z
Tavares et al. 2013	13.2, 15.1, 45.5, 74.2	Batch broth/plates (DSM or F medium)	doi.org/10.1007/s00284-012-0269-2
Widderich et al. 2015	0, 0.5, 1, 40, 70, 90	Batch DSM cultures (nutrient exhaustion, ±NaCl)	doi.org/10.1111/mmi.13304
Méndez et al. 2004	0.0025, 0.003, 0.0079, 0.014, 0.0213, 0.1	Batch (MM or Schaeffer, shaking flasks)	doi.org/10.1128/JB.186.4.989-1000.2004

Table S3. Definitions, data sources, and calculation methods for energetic cost comparisons (Figure 3).

Trait/Process	Definition & Duration	Data Sources, Calculation Method & Key Assumptions
**Spore life cycle**	Complete developmental program: sporulation (8 h), germination (0.25 h), outgrowth (3.25 h). Total: 11.5 h at 20°C (∼10 vegetative generations).	*Data sources:* Time-resolved expression from SporeWeb(14, 15) (sporulation), Swarge et al.(16) (germination/outgrowth). Protein abundances from PaxDB(17, 18). Gene/protein metadata from SubtiWiki(19). *Calculation:* Build costs (transcription and translation) integrated over developmental time points. For each gene j: (1) Transcription cost = gene length × transcript number × per-nucleotide cost (synthesis + polymerization); (2) Translation cost = protein copies × per-amino-acid cost. Expression weights ($w_{j,t}$) distribute costs across hours (Eqs. 7–11). *Figure 3 bar:* Build costs only = 7.8×10^9 ATP. *Total spore generation cost* (including genome replication + septum + membrane remodeling) = 9.2×10^9 ATP (∼10% of cellular budget). *Key assumption:* Sporulation is a regulated developmental program with stage-specific gene expression(20), requiring temporal integration of costs across the 11.5 h cycle. Time-resolved datasets enable this approach. Protein turnover is excluded to avoid double-counting with maintenance.
**Maintenance energy**	Continuous operating cost for core housekeeping metabolism: ion gradients, minimal turnover, osmoregulation, redox balance. Not trait-specific.	*Calculation:* $C_M \times t_{\text{gen}} = 1.16 \times 10^9$ ATP $h^{-1} \times 1.16$ h $= 1.3 \times 10^9$ ATP per vegetative generation. C_M from Lynch & Marinov(21). *Key distinction:* Maintenance is an **operating rate** (ATP/hour), while homeostasis is a **build cost** (one-time synthesis).
**Homeostasis**	Stress-response machinery: chaperones, proteases, antioxidants. Primarily σ^B-regulated(22).	*Data sources:* Gene list from SubtiWiki "Homeostasis" category. Protein abundances from PaxDB (steady-state, exponential growth). *Calculation:* One-time build cost. Total = 1.4×10^9 ATP. *Key assumptions:* (1) Snapshot from bulk proteomics; no temporal dynamics. (2) Represents build cost assuming per-generation resynthesis for conservative comparison. (3) Homeostasis quantifies the **one-time build cost** of stress-response machinery, while maintenance energy quantifies the **continuous operating rate** (ATP/hour) required for core housekeeping functions.

**Heat shock proteins**	Molecular chaperones and proteases (DnaK, GroEL, ClpP family). Induced under thermal/protein-folding stress.	*Data sources:* Gene list from SubtiWiki. Abundances from PaxDB.<br>*Calculation:* Build cost assuming per-generation resynthesis. Total = 1.2×10^9 ATP.<br>*Key assumptions:* (1) Snapshot from bulk proteomics; no temporal dynamics. (2) Represents build cost assuming per-generation resynthesis for conservative comparison. (3) Overlap with homeostasis category (e.g., DnaK, GroEL).
**Flagellum**	Peritrichous flagellar apparatus: basal body, hook, filament (flagellin). *B. subtilis* assembles ∼26 flagellar basal bodies per cell(23). Assembly: ∼40–60 min(23, 24).	*Data sources:* Flagellar genes (*fli*, *flg*, *fla* operons) from SubtiWiki. Abundances from PaxDB.<br>*Calculation:* Build cost assuming complete per-generation resynthesis.<br>*Key assumptions:* (1) Conservative estimate: We assume complete per-generation resynthesis of all flagellar structures. However, flagella are stable structures that persist through cell division and are inherited by daughter cells(23). During division, existing flagella are distributed between daughters (sometimes unequally), reducing the need for complete resynthesis each generation. True per-generation costs are therefore likely lower than reported here, further emphasizing the relative expense of sporulation. (2) Build cost only; operating costs excluded. The bacterial flagellar motor is driven by proton flux across the membrane. For *E. coli*, rotation requires ∼1,100 H^+ per revolution(25). At 200 Hz, this corresponds to $\sim 6.6 \times 10^4$ ATP s^{-1} per flagellum. For a cell with 3.4 flagella operating continuously for one generation (1.16 h), the total operating cost $\sim 8.1 \times 10^8$ ATP(25), comparable to the build cost. Operating costs scale with rotation rate and flagella number; intermittent swimming reduces the burden. (3) Snapshot from steady-state growth.
**Chemotaxis**	Signal transduction for directed motility: Methyl-accepting chemotaxis proteins, Che proteins(26, 27). Active during vegetative growth.	*Data sources:* *che* operon genes, MCPs (mcpA/B/C, tlpA/B/C), regulators (sigD, swrA/B/D) from SubtiWiki. Abundances from PaxDB.<br>*Calculation:* One-time build cost for complete chemotaxis machinery.<br>*Key assumption:* (1) Potential operational costs such as CheA autophosphorylation, receptor methylation/demethylation(26) are not included. These ongoing signaling costs are small relative to build costs. (2) Assumes per-generation resynthesis for conservative comparison, though some structural components may persist across divisions.
**Swarming**	Multicellular surface motility requiring flagella and surfactin production(27).	*Data sources:* Combined flagellar, chemotaxis, and surfactin biosynthesis genes from SubtiWiki. Abundances from PaxDB.<br>*Calculation:* One-time build cost for complete swarming machinery.<br>*Key assumptions:* (1) Excludes active swimming costs and surfactin secretion energy. (2) Assumes per-generation resynthesis for conservative comparison, though some structural components may persist across divisions.

**Biofilm**	Matrix-encased communities: exopolysaccharides (Eps), TasA protein, TapA amyloid, eDNA(28, 29). Pellicle formation: 8–12 h; mature by 12–24 h(30).	*Data sources:* Matrix genes (*eps*, *tasA*, *tapA*, *bslA* operons) from SubtiWiki. Abundances from PaxDB.<br>*Calculation:* Build cost from steady-state abundances, divided by program duration ($\sim$12 h $\approx$ 10.3 vegetative generations) to obtain per-generation equivalent.<br>*Key assumptions* (1) Only $\sim$10% of cells are matrix producers. Bulk proteomics averages across producers and non-producers. Value represents population-averaged cost; per-producer costs are likely 10× higher. (2) No temporal dynamics of matrix gene expression during a 12 h biofilm program. Cannot time-integrate like the spore cycle, so we divide the total estimated cost by the duration to obtain a per-generation-time equivalent. (3) Excludes metabolic costs such as acetoin production (57% of glycerol consumed), TCA cycle upregulation, nucleotide biosynthesis for eDNA, bacillibactin production(29).
**Competence**	Genetic transformation machinery. Transient K-state: $\sim$3 h during late exponential phase(31).	*Data sources:* Competence genes (*com* operons) from SubtiWiki. Abundances from PaxDB.<br>*Calculation:* Build cost from steady-state abundances, divided by program duration ($\sim$3 h $\approx$ 2.6 vegetative generations) to obtain per-generation equivalent.<br>*Key assumption:* Only $\sim$10–20% of cells become competent. Bulk measurements yield population-averaged costs. Per-competent-cell costs likely 5–10× higher.
**Essential genes**	Core viability machinery: ribosomes, RNA polymerase, central metabolism, cell division(19).	*Data sources:* Genes identified as essential from knockout studies (SubtiWiki). Abundances from PaxDB.<br>*Calculation:* One-time build cost assuming per-generation resynthesis.<br>*Key assumption:* Conceptual overlap with maintenance (both represent core cellular functions), but essential genes quantify structural synthesis cost, while maintenance quantifies ongoing metabolic rate.
**Genome replication**	Complete chromosome duplication (4,215,606 bp, NCBI NC_000964.3). Once per cell division.	*Calculation:* Direct costs: polymerization (2 ATP/nt), helicase (1 ATP/bp), Okazaki primers (0.32 ATP/bp)(21). Opportunity costs: dNTP precursor synthesis ($\sim$34 P_O/nt)(32).<br>Total: $C_R = 2L_g(c_s + c_p) + L_g(c_{\text{hel}} + c_{\text{prim}}) \approx 4.0 \times 10^8$ ATP. Factor of 2 for bidirectional replication of both strands(Eq. 5–6).
**Membrane lipids**	Complete cellular bilayer. Area from cell geometry (spherocylinder: $L = 2.5$ μm, $D = 1.0$ μm)(33).	*Data sources:* Cell dimensions from Barak et al.(33). Lipid costs from Mahmoudabadi et al.(34). Membrane parameters from Nagle & Tristram-Nagle(35), Petrache et al.(36), Lewis & Engelman(37), Mitra et al.(38).<br>*Calculation:* Lipid number = bilayer area / head-group area ($a_1 = 0.65$ nm^2)(35, 36). Cost per lipid: 18 P_D + 212 P_O(34). Bilayer thickness $h = 4$ nm(37, 38). Assumes 50% protein occupancy; lipid-only area = 0.5 × total area (Eq. 12–14).
**Total cell budget**	Reference: total per-generation energy at 20°C ($t_{\text{gen}} = 1.16$ h).	*Data sources:* Growth cost (C_G) and maintenance rate (C_M) from Lynch & Marinov(21).<br>*Calculation:*<br>$C_T = C_G + (t_{\text{gen}} \times C_M) = 9.25 \times 10^{10} + 1.3 \times 10^9 \approx 9.4 \times 10^{10}$ ATP per generation.<br>C_G = per-generation growth/build cost. C_M = per-hour maintenance rate.

Table S4. Representative frameworks for cellular and molecular bioenergetic accounting and their implications for this study.

Reference / Framework	Approach	Estimated total ATP per division	Key assumptions	Effect on this study
Lynch & Marinov (2015)	Empirical–theoretical, bottom-up bioenergetic accounting that combines chemostat-based ATP budgets with biochemical cost models for nucleotides and amino acids.	10^9–10^{10} ATP cell^{-1} generation^{-1}	Growth and maintenance costs are empirically derived from measured ATP fluxes; per-gene costs are computed from the stoichiometric ATP or GTP requirements of nucleotide and amino-acid synthesis, transcription, and translation.	Forms the empirical baseline adopted here; all trait-level ATP costs are expressed relative to this framework.
Mahmoudabadi et al. (2017)	Molecular-to-cellular bottom-up accounting of viral life cycles; explicit summation of transcriptional, translational, and assembly ATP costs.	$\sim 10^9$–10^{10} ATP infection^{-1} (T4 phage $\approx 10^{10}$)	Introduces the distinction between direct and opportunity costs (E_D, E_O); total cost $E_T = E_D + E_O$ defines full metabolic demand of macromolecular synthesis.	Conceptually closest precedent to this work; our trait-level bioaccounting extends this framework from viral replication to bacterial sporulation.
Lane & Martin (2010, 2016)	Theoretical, supply-limited framework emphasizing constraints from bioenergetic membrane area and mitochondrial amplification.	10^{11}–10^{12} ATP cell^{-1} generation^{-1}	ATP supply per gene scales with membrane surface area; mitochondria enable large increases in energy per gene.	Defines the upper bound of possible cellular energy budgets; shifting to this scale raises absolute costs by 10–100X. Not applicable to prokaryotic physiology, which lacks mitochondria and does not experience membrane-area–limited ATP supply.
Lane (2011); Lane (2020)	Conceptual synthesis extending the supply-limited argument to genome expansion and cellular complexity.	Same as above (10^{11}–10^{12})	Energy flow and respiratory membrane topology shape evolutionary potential; mitochondria create energetic asymmetry.	Cited to frame the broader evolutionary debate over supply- versus demand-driven energetic constraints.
This study (trait-level bottom-up bioaccounting)	Summation of gene and protein ATP expenditures for sporulation and comparison traits, anchored to the empirical per-generation total.	1.3×10^9 (range 10^9–10^{11}) ATP cell^{-1} generation^{-1}	Assumes demand-based ATP budgeting; integrates macromolecular synthesis and maintenance costs at the trait level.	Varying total ATP budgets across the published range shifts crossover times by ¡2×; qualitative conclusions unchanged.

References

1. Z Wang, et al., Complementary resource preferences spontaneously emerge in diauxic microbial communities. *Nat. Commun.* **12**, 6661 (2021) Publisher: Nature Publishing Group.
2. Y Fridman, Z Wang, S Maslov, A Goyal, Fine-scale diversity of microbial communities due to satellite niches in boom and bust environments. *PLOS Comput. Biol.* **18**, e1010244 (2022) Publisher: Public Library of Science.
3. A Goyal, G Chure, Paradox of the Sub-Plankton: Plausible Mechanisms and Open Problems Underlying Strain-Level Diversity in Microbial Communities. *Environ. Microbiol.* **27**, e70094 (2025) _eprint: https://enviromicro-journals.onlinelibrary.wiley.com/doi/pdf/10.1111/1462-2920.70094.

4. AC Fowler, HF Winstanley, Microbial dormancy and boom-and-bust population dynamics under starvation stress. *Theor. Popul. Biol.* **120**, 114–120 (2018).
5. AV Narla, T Hwa, A Murugan, Dynamic coexistence driven by physiological transitions in microbial communities. *Proc. Natl. Acad. Sci.* **122**, e2405527122 (2025) Publisher: Proceedings of the National Academy of Sciences.
6. PY Ho, BH Good, KC Huang, Competition for fluctuating resources reproduces statistics of species abundance over time across wide-ranging microbiotas. *eLife* **11**, e75168 (2022) Publisher: eLife Sciences Publications, Ltd.
7. J Lin, M Manhart, A Amir, Evolution of Microbial Growth Traits Under Serial Dilution. *Genetics* **215**, 767–777 (2020).
8. JW Fink, NA Held, M Manhart, Microbial population dynamics decouple growth response from environmental nutrient concentration. *Proc. Natl. Acad. Sci.* **120**, e2207295120 (2023) Publisher: Proceedings of the National Academy of Sciences.
9. D Cohen, Fitness in Random Environments in *Adaptation in Stochastic Environments*, eds. J Yoshimura, CW Clark. (Springer, Berlin, Heidelberg), pp. 8–25 (1993).
10. A Măgălie, DA Schwartz, JT Lennon, JS Weitz, Optimal dormancy strategies in fluctuating environments given delays in phenotypic switching. *J. Theor. Biol.* **561**, 111413 (2023).
11. M Abramowitz, IA Stegun, *Handbook of Mathematical Functions: With Formulas, Graphs, and Mathematical Tables.* (Courier Corporation), (1965) Google-Books-ID: MtU8uP7XMvoC.
12. IW Dawes, J Mandelstam, Sporulation of *Bacillus subtilis* in Continuous Culture. *J. Bacteriol.* **103**, 529–535 (1970).
13. P Virtanen, et al., SciPy 1.0: fundamental algorithms for scientific computing in Python. *Nat. Methods* **17**, 261–272 (2020) Publisher: Nature Publishing Group.
14. RT Eijlander, A de Jong, AO Krawczyk, S Holsappel, OP Kuipers, SporeWeb: an interactive journey through the complete sporulation cycle of Bacillus subtilis. *Nucleic Acids Res.* **42**, D685–D691 (2014).
15. P Nicolas, et al., Condition-Dependent Transcriptome Reveals High-Level Regulatory Architecture in Bacillus subtilis. *Science* **335**, 1103–1106 (2012).
16. B Swarge, et al., Integrative Analysis of Proteome and Transcriptome Dynamics during Bacillus subtilis Spore Revival. *mSphere* **5** (2020) Publisher: American Society for Microbiology Journals Section: Research Article.
17. M Wang, et al., PaxDb, a Database of Protein Abundance Averages Across All Three Domains of Life*. *Mol. & Cell. Proteomics* **11**, 492–500 (2012).
18. M Wang, CJ Herrmann, M Simonovic, D Szklarczyk, Cv Mering, Version 4.0 of PaxDb: Protein abundance data, integrated across model organisms, tissues, and cell-lines. *Proteomics* **15**, 3163 (2015) Publisher: Wiley.
19. B Zhu, J Stülke, SubtiWiki in 2018: from genes and proteins to functional network annotation of the model organism Bacillus subtilis. *Nucleic Acids Res.* **46**, D743–D748 (2018).
20. DW Hilbert, PJ Piggot, Compartmentalization of Gene Expression during Bacillus subtilis Spore Formation. *Microbiol. Mol. Biol. Rev.* **68**, 234–262 (2004).
21. M Lynch, GK Marinov, The bioenergetic costs of a gene. *Proc. Natl. Acad. Sci.* **112**, 15690–15695 (2015) Publisher: National Academy of Sciences Section: Biological Sciences.
22. F Rodriguez Ayala, M Bartolini, R Grau, The Stress-Responsive Alternative Sigma Factor SigB of Bacillus subtilis and Its Relatives: An Old Friend With New Functions. *Front. Microbiol.* **11** (2020).
23. S Mukherjee, DB Kearns, The structure and regulation of flagella in Bacillus subtilis. *Annu. review genetics* **48**, 319–340 (2014).
24. DF Blair, HOW BACTERIA SENSE AND SWIM. *Annu. Rev. Microbiol.* **49**, 489–520 (1995) Publisher: Annual Reviews.
25. PE Schavemaker, M Lynch, Flagellar energy costs across the tree of life. *eLife* **11**, e77266 (2022).
26. V Sourjik, NS Wingreen, Responding to Chemical Gradients: Bacterial Chemotaxis. *Curr. Opin. Cell Biol.* **24**, 262–268 (2012).
27. R Colin, B Ni, L Laganenka, V Sourjik, Multiple functions of flagellar motility and chemotaxis in bacterial physiology. *FEMS microbiology reviews* **45**, fuab038 (2021).
28. H Vlamakis, Y Chai, P Beauregard, R Losick, R Kolter, Sticking together: building a biofilm the Bacillus subtilis way. *Nat. Rev. Microbiol.* **11**, 157–168 (2013) Publisher: Nature Publishing Group.
29. T Pisithkul, et al., Metabolic Remodeling during Biofilm Development of Bacillus subtilis. *mBio* **10**, e00623–19 (2019).
30. M Krajnc, et al., Systems view of Bacillus subtilis pellicle development. *npj Biofilms Microbiomes* **8**, 25 (2022) Publisher: Nature Publishing Group.
31. D Dubnau, Genetic competence in Bacillus subtilis. *Microbiol. Rev.* **55**, 395–424 (1991).
32. G Mahmoudabadi, R Phillips, M Lynch, R Milo, Defining the Energetic Costs of Cellular Structures, (Biophysics), preprint (2019).
33. I Barák, K Muchová, The positioning of the asymmetric septum during sporulation in Bacillus subtilis. *PLoS ONE* **13**, e0201979 (2018).
34. G Mahmoudabadi, R Milo, R Phillips, Energetic cost of building a virus. *Proc. Natl. Acad. Sci.* **114**, E4324–E4333 (2017).
35. JF Nagle, S Tristram-Nagle, Lipid bilayer structure. *Curr. Opin. Struct. Biol.* **10**, 474–480 (2000).
36. HI Petrache, SW Dodd, MF Brown, Area per Lipid and Acyl Length Distributions in Fluid Phosphatidylcholines Determined by 2H NMR Spectroscopy. *Biophys. J.* **79**, 3172–3192 (2000).
37. BA Lewis, DM Engelman, Lipid bilayer thickness varies linearly with acyl chain length in fluid phosphatidylcholine vesicles. *J. Mol. Biol.* **166**, 211–217 (1983).
38. K Mitra, I Ubarretxena-Belandia, T Taguchi, G Warren, DM Engelman, Modulation of the bilayer thickness of exocytic pathway membranes by membrane proteins rather than cholesterol. *Proc. Natl. Acad. Sci.* **101**, 4083–4088 (2004) Publisher: Proceedings of the National Academy of Sciences.
